# The Ohio Dental College Association

**Published:** 1865

**Authors:** 


### THE OHIO DENTAL COLLEGE ASSOCIATION.

The proceedings of this association, held on the 21, 22 and 23 of February last, are published in this number and render any extended notice here unnecessary.

This meeting of the stockholders was one of the largest that has ever been held. A great degree of interest was manifested in the welfare of the College, and the utmost unanimity prevailed in the deliberations. The subject of a reorganization came up and was freely discussed. The chief points in that matter were that there should be rotation in the board of trustees, and that it should be composed principally of members of the profession, who feel an interest in and are acquainted with the wants of the profession. In order to accomplish this some change was necessary to be made in the charter. Accordingly an applicantion was made to the legislature, by a committee appointed for the purpose.

A bill to regulate Colleges of Dental Surgery was passed, which embraced the desired points. Under this law the College will be organized and act, at and after the next annual meeting of the association. The following is a copy of the Act.

